# Correction: Trends in clinical characteristics and outcomes of all critically ill COVID-19 adult patients hospitalized in France between March 2020 and June 2021: a national database study

**DOI:** 10.1186/s13613-023-01111-2

**Published:** 2023-03-19

**Authors:** Diane Naouri, Albert Vuagnat, Gaëtan Beduneau, Martin Dres, Tai Pham, Alain Mercat, Alain Combes, Alexandre Demoule, Antoine Kimmoun, Matthieu Schmidt, Matthieu Jamme

**Affiliations:** 1 Department for Research, Studies, Assessment and Statistics (DREES), French Ministry of Health, 10 Place Des 5 Martyrs du Lycée Bufon, 75014 Paris, France; 2 UNIROUEN, EA 3830, Medical Intensive Care Unit,, Rouen University Hospital, Normandie University, 76000 Rouen, France; 3 Service de Pneumologie et Réanimation Médicale, Hôpital Pitié Salpétrière, Assistance Publique Hôpitaux de Paris, Paris, France; 4 Service de Médecine Intensive-Réanimation, Hôpital du Kremlin Bicêtre, Assistance Publique Hôpitaux de Paris, Le Kremlin Bicêtre, France; 5 Service de Réanimation Médicale et Médecine Hyperbare, CHU Angers, Angers, France; 6 Sorbonne Université, GRC 30, RESPIRE, UMRS_1166-ICAN, Institute of Cardiometabolism and Nutrition, Service de Médecine Intensive-Réanimation, Assistance Publique-Hôpitaux de Paris (APHP) Hôpital Pitié-Salpêtrière, Paris, France; 7 Service de Médecine Intensive-Réanimation, CHRU Nancy, Nancy, France; 8 Service de Réanimation Polyvalente, Hôpital Privé de l’Ouest Parisien, Ramsay-Générale de Santé, Trappes, France; 9 CESP, INSERM U1018, Equipe Epidémiologie Clinique, Villejuif, France

**Correction: Annals of Intensive Care (2023) 13:2** **https://doi.org/10.1186/s13613-022-01097-3**

In the original publication [1] of the article, the Fig. 2 was incorrectly published where the line from Fig. 2B have been moved to Fig. 2A by mistake. The corrected Fig. 2 is given in this Correction article. The original article has been corrected.

**Fig. 2:**
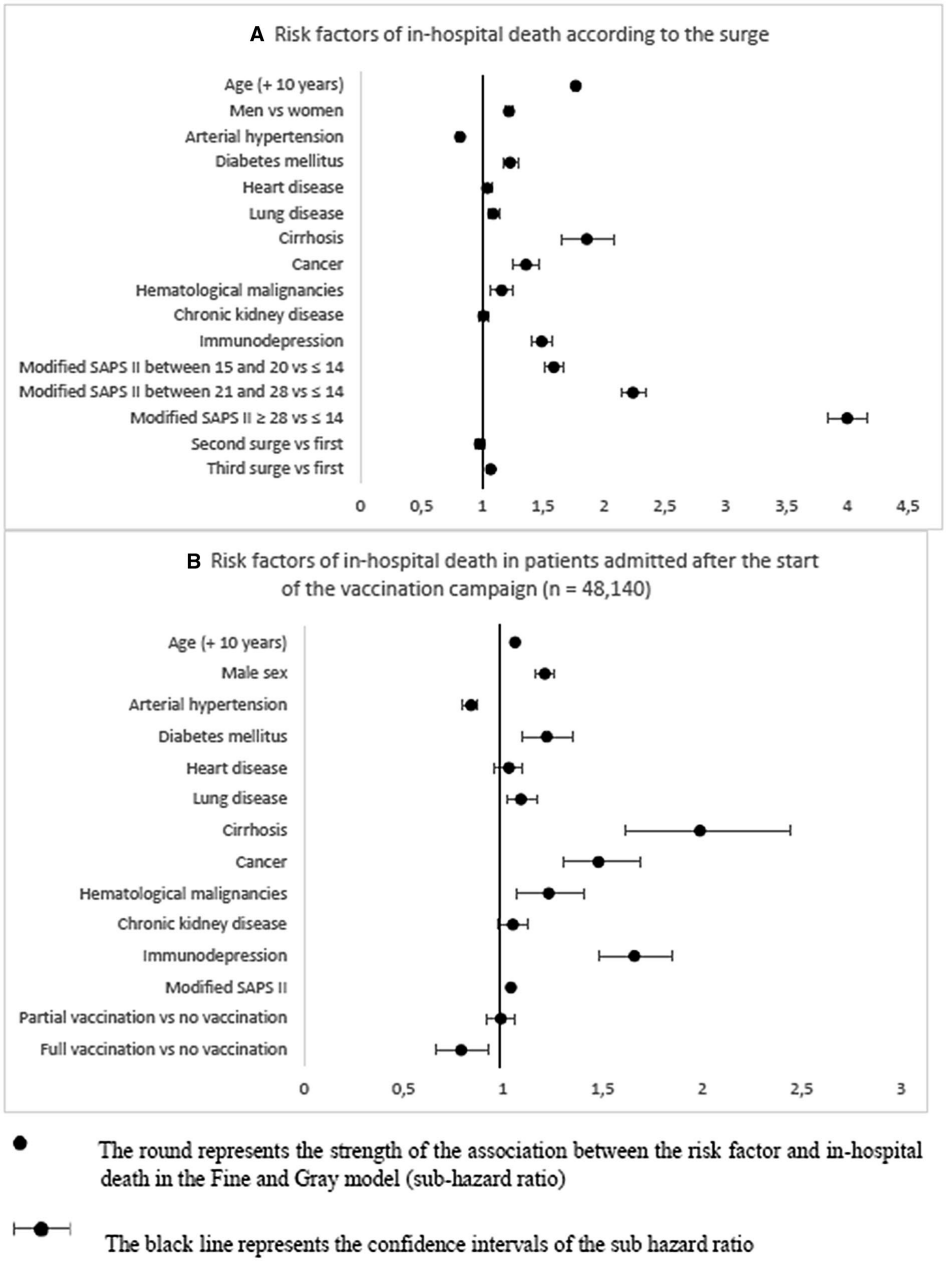
Risk factors of in-hospital death
